# Quick Analysis of the Influence of the Monsoon on the Concentration of Inorganic Anthropogenic Particles in the Air

**DOI:** 10.1007/s00128-026-04223-0

**Published:** 2026-03-11

**Authors:** Cecile de Souza Gama, Gabriela Gama Jaster, Amanda Soares Arnaud, Jefferson Erasmo de Souza Vilhena, Luis Maurício Abdon da Silva

**Affiliations:** 1 Department of Ichthyology, The Academy of Natural Sciences of Drexel University, Philadelphia, PA 19103 USA; 2 Laboratório de Ictiologia, Núcleo de Biodiversidade, Instituto de Pesquisas Científicas e Tecnológicas do Estado do Amapá, Macapá, AP Brazil; 3 Faculdade de Ciências Biológicas, Universidade Federal do Amapá, Macapá, AP Brazil; 4 Núcleo de Hidrometeorologia e Energias Renováveis (NHMET), Instituto de Pesquisas Científicas e Tecnológicas do Estado do Amapá, Macapá, AP Brazil; 5 Laboratório de Biologia e Dinâmica de Recursos Pesqueiros, Núcleo de Pesquisas Aquáticas, Instituto de Pesquisas Científicas e Tecnológicas do Estado do Amapá, Macapá, AP Brazil

**Keywords:** Anthropogenic particles pollution, Rainwater, Rainy periods, Amazonic monsoon

## Abstract

Anthropogenic particles occur in various aquatic environments, and rainwater acts as a carrier of these contaminants by incorporating them into water droplets. To test the potential of rainfall to remove airborne particles, rainwater was collected three times during the rainy season in the municipality of Macapá, Amapá, Brazil. Chemical digestion and filtration protocols were applied to identify particles. A total of 225 ml of rainwater yielded 194 particles. Particle abundance increased with the number of preceding rain-free days, suggesting that rainfall may contribute to the atmospheric removal of these particles.

## Introduction

Anthropogenic particles (organic and inorganic) can be found in a wide range of sizes, and those on the microscale and nanoscale are currently receiving attention from society and governments around the world because of the increased awareness of their existence and potentially harmful consequences (Mattsson et al. 2021). Engineered nanomaterials, microplastics and nanoplastics, soot, road and tire wear are a few prominent examples of particles that are either intentionally manufactured or incidentally produced and released into the environment (Mattsson et al. 2021). The percentage of microplastics in the air is comparatively higher than in any other medium, which indicates that all humans are susceptible to inhaling such harmful particles. However, monitoring the abundance of these particles in certain areas is considered insufficient in assembling preventative measures that reduce and prevent microplastics pollution because the particles travel globally through various routes (Ross et al. 2021).

Microplastics, a component of anthropogenic particles, are not inert (Illuminati et al. 2024); they react with atmospheric oxidants like oxygen, ozone, hydroxyl radicals, and nitrogen oxides (Bianco et al. 2020; Vicente et al. 2009), producing harmful organic compounds. These compounds pose risks to ecosystems and can accumulate in aquatic organisms, causing internal injuries, and behavioral changes (Cole et al. 2011). When it rains, airborne particles are collected and concentrated by rain droplets and become what is so-called “plastic rain” (Brahney et al. 2020). Pignattelli et al. (2021) demonstrated that microplastics toxicity may increase when coupled with acid rain. This study focuses on the atmospheric component of anthropogenic inorganic particles and investigates whether rain can act to remove this pollutant from the air.

## Methods and Materials

Following the protocol of Do et al. (2023), rainwater was collected during the monsoon in three rainfall days (June and July 2024) in an environment with low urban density (0°1′ 26.43″ S, 51°4′ 41.38″ W) (Fig. SI-1) near the Amazon River in the municipality of Macapá, state of Amapá, Brazil. Meteorological data for the collection days (precipitation, average daily temperature, maximum relative air humidity) were provided by the Center for Hydrometeorology and Renewable Energy (NHMET), Institute of Scientific and Technological Research of the State of Amapá, Macapá, AP, Brazil.

The atmospheric precipitation was collected during the first 15 min of discharge (first-flush effect (Martinson and Thomas 2005)) by standardized glass containers with a mouth diameter of 60 mm and previously washed with distilled water. As proposed by Do et al. (2023), the glass containers were placed one-meter above the surface to prevent additional input of particles from the ground surface. The amount of water collected ranged from 55 to 100 mL according to the intensity of the rain.

The collected water was transferred to sterile beakers and, to remove organic matter, enough KOH was added to form a 10% solution. The beakers were covered with aluminum foil and heated at 60 °C for 24 h (Suwartiningsih et al. 2020). After digestion, the remaining solution was poured through filters (pore size 50 μm), placed onto sterile petri dishes for stereomicroscope examination of inorganic particles (Olympus SZ61 Stereo Microscope, 45x) and categorized by color, size and shape (GESAMP 2019) and measured with a ruler under the microscope. Once the entire chemical digestion process is completed, only inorganic matter is expected to remain in the final solution. Therefore, we assumed that all particles found were inorganic anthropogenic particles (IAP), since Fourier transform infrared spectroscopy (FTIR) or Raman spectroscopy was not conducted to confirm their polymer composition.

All laboratory procedures involved necessary precautions to avoid possible contamination of the samples as done in Miranda et al (2025), including control blanks. Detailed information is provided in the Supplementary Material.

To standardize the results across samples, we report IAPs per 100 mL of rainwater collected (Table 1). The data were analyzed using descriptive statistics as well as a Spearman correlation analysis between the amount of IAP and precipitation (Siegel 1975).

**Table 1:** IAPs found in the collections and respective meteorological data

Samples	IAP	ml	AIP/100 ml	PP (mm^3^)	TA (°C)	RAH max	Previous PP
1. 1 June	69	100	69	27,6	29	94	1,2 mm on 31 May (day before)
2. 24 July	82	70	117	12,4	28	90	1,2 on 21 June (3 days before)
3. 30 July	43	55	78	7,4	29	90	30,2 mm on 29 July (day before)

where PP – Precipitation; AT – Average daily temperature; RAHmax – maximum relative air humidity

## Results and Discussion

We found a total of 194 inorganic anthropogenic particles (IAP) in 225 mL of total analyzed rainwater, corresponding to 0.86 IAP per milliliter and an average of 64.6 particles per day of collection. These results revealed that a rain event washes out IAPs from the atmosphere. Regarding the type of IAP found, 96.9% were classified as fibers, followed by pellets (2.6%) and 1 foam particle (0.5%). Ross et al. (2023) also found fibers to be the most abundant particles in their work with microplastics on rainwater in Alberta, Canada. Dris et al. (2016) similarly found a significant number of fibers in atmospheric fallout in urban areas of France, and 29% of those fibers contained petrochemicals. Martinson and Thomas (2005) explained that the initial rainwater during the rain event (first-flush phenomenon) can remove up to 85% of particles accumulated on the ground surface. Thus, the airborne IAPs flushed by rainwater and the IAP already on the ground are both drained into water bodies during rain events.

Most fibers found were black (54%), followed by blue (25%), red (13%), green (9%) and pink (6%). These colors and their proportions are generally like those found in aquatic environments or in the gastrointestinal tract of fishes (Miranda et al. 2025; Aunurohim et al. 2023; Khan and Setu 2022; Andrade et al. 2019) indicating that air may be an important source of IAP that reach these environments, while for the air, Welsh et al. (2022) found predominance of blue and red.

Our results constitute a snapshot of the atmospheric concentration of IAPs at the time of sampling. Since rainwater washes the air, IAPs are expected to accumulate in the air over time between rains. Thus, more particles are expected in rain events that occur over longer periods of dryness. Our results were consistent with this (Table 1), as we found a greater number of IAPs in rainwater after two dry days vs. rain on the day before sampling occurred. This suggests that atmospheric IAPs contamination is constant because IAPs were found in the following day’s rain, however, in smaller quantities. In this way, the rainy season (moonson) will contribute to cleaning IAP from the air but will increase the arrival of this contaminant in the bodies of water that receive this flow.

**Fig. 1:**
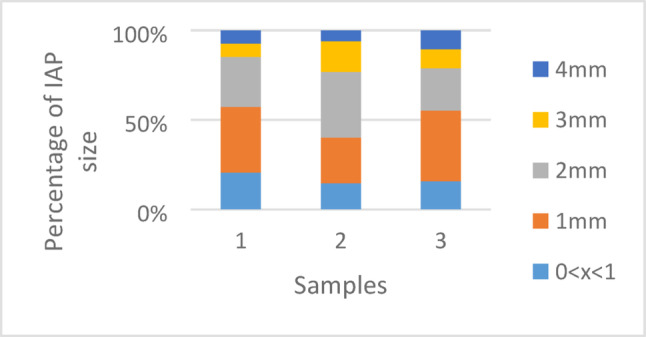
Size distribution of IAPs particles during the three samples

We found a predominance of 1- and 2-mm fibers in the rainwater. The size distribution of the fragmented IAPs showed a higher number of smaller particles (Fig. 1). This greater number of smaller particles, as also noted by Dris et al. (2016), with microplastics in the air likely because larger particles are more easily captured during the first 10 min of a rain event (Abbasi 2021), and also because the atmospheric floating time (air suspension) increases for smaller particles according to Stokes’ law (Van Sebille et al. 2020), so probably we do have more smaller particles in the air. Furthermore, as plastic particle size decreases, they release more mobile and bioavailable chemicals (MacLeod et al. 2021), leading to more severe and poorly reversible pollution than that produced when they first entered the environment (Wang et al. 2025). The aerodynamic shape of particles is also important for understanding atmospheric residence time but is still poorly understood for fibers and other asymmetric shapes (Brahney et al. 2020). We found no relationship between the amount of IAP found and the daily temperature or relative humidity probably due to the small sample size. These factors possibly affect the persistence of particles in the air, and then longer studies are needed to establish this possible relationship.

This study provides the first data on the presence of anthropogenic inorganic particles in the atmosphere of the Amazon region, particularly in the state of Amapá (Brazil), where information remains scarce. The findings offer baseline evidence and highlight the need for further systematic investigations in this environmentally sensitive region.
